# Cash transfer, maternal and child health outcomes: a scoping review in sub-Saharan Africa

**DOI:** 10.1080/16549716.2024.2309726

**Published:** 2024-02-09

**Authors:** Emery L. Ngamasana, Jessamyn Moxie

**Affiliations:** Department of Public Health Sciences, College of Health and Human Services, University of North Carolina at Charlotte, Charlotte, NC, USA

**Keywords:** Cash transfer, health outcomes, International Classification of Functioning, disability and health, maternal and child health

## Abstract

**Background:**

Cash Transfer (CT) programmes can improve maternal and child health outcomes in low- and middle-income countries. However, studies assessing the effectiveness of these programmes on maternal and child health outcomes (MCH), beyond nutritional outcomes and service utilisation, remain inconclusive.

**Objectives:**

We synthesized current empirical evidence on the effectiveness of these programmes in improving MCH outcomes and suggested a framework for reporting such outcomes. We focused on sub-Saharan Africa because of substantial operational differences between regions, and the need for MCH advancement in this region.

**Methods:**

This review searched PubMed Central and Google Scholar and supplemented it with a backward citation search for studies conducted in sub-Saharan Africa for the period between 2000 and 2021. Only peer-reviewed studies on CT that reported health outcomes beyond nutritional outcomes and service utilisation among women of reproductive age and children below 18 years old were included.

**Results:**

Twenty-one articles reporting studies conducted in six sub-Saharan African countries were identified. All studies reported health outcome measures, and programmes targeted women of reproductive age and children under 18 years of age. Of the 21 articles, 1 reported measures of mortality, 13 reported measures of functional status; 3 reported subjective measures of well-being, and 4 reported behavioural health outcomes. Across all categories of reported measures, evidence emerges that cash transfer programmes improved some health outcomes (e.g. improved infant and child survival, reduced incidence of illnesses, improved cognitive and motor development, improved general health, delayed sexual debut, lower transactional sex, etc.), while in some of the studies, outcomes such as depression did not show significant improvements.

**Conclusion:**

Cash Transfer programmes are effective and cost-effective, with a real potential to improve maternal and child health outcomes in sub-Saharan African countries. However, further research is needed to address implementation challenges, which include data collection, and programme management.

## Introduction

Populations in Low- and Middle-Income Countries (LMIC) continue to face daunting challenges. Mothers and children are the most affected by these challenges, which include a double burden of communicable and non-communicable diseases, higher infant and maternal mortality, malnutrition, etc. These challenges have been exacerbated, by the novel COVID-19 pandemic, which has dismantled the prevailing social safety nets [1–3]. The future remains ambiguous, with vaccine access as the fault line along which economic recovery may split [4].

Cash Transfer (CT) programmes are one of the social protection and poverty alleviation interventions that policymakers and funders can rely on to improve MCH outcomes in LMICs. These programmes were initially launched in Latin America (i.e. Brazil and Mexico) in the mid-90s to provide education and health to historically ‘excluded’ populations before being adopted in other LMICs [5].

CT programmes represent a ‘set of public and private policies or programmes aimed at preventing, reducing and eliminating economic and social vulnerability to poverty and deprivation’ [6]. They are often implemented as a direct transfer payment of money to eligible persons and can be either conditional or unconditional. Unconditional Cash Transfer (UCT) programmes provide cash to all eligible and registered beneficiaries; whereas Conditional Cash Transfer (CCT) programmes require an eligible person to take a specified course of action, also known as co-responsibilities or conditionalities, to receive a benefit [7,8]. Generally, co-responsibilities include actions such as attending required medical check-ups, completing required immunisations, school attendance, and adults attending education seminars covering topics such as health, family planning, nutrition, adherence to immunisation, registering childbirth, exclusive breastfeeding, etc [8,9].

These programmes have been widely implemented in Latin America, Africa, and South Asia. For instance, Bolsa Familia introduced in Brazil in 2003 after merging three existing conditional and unconditional cash transfer programmes, is one of the largest CCT programmes in the world [10,11]. It covered over 14 million eligible households that met the eligibility criteria: households with children less than 17 years of age and/or pregnant women making less than R$120 (USD 68) per capita monthly. In addition to this overall payment, the Bolsa Familia programme also sent monthly transfers to extremely poor families (i.e. those earning less than R$64 or USD 34), regardless of their composition [12].

CT programmes are also common among Asian countries. For instance, in 2011 the state of Odisha in India initiated a statewide CCT programme named Mamata to improve MCH outcomes and promote health-seeking behaviors [9]. Other similar programmes in South Asia include the Aama (Mothers’) programme in Nepal, the Janani Suraksha Yojana (Safe Motherhood Scheme) and the Chiranjeevi Yojana (Scheme for Long Life) in India; the Maternal Health Voucher Scheme in Bangladesh and the Sehat (Health) Voucher Scheme in Pakistan [13].

In Africa, major CT programmes such as the Productive Safety Net Programme’s Direct Support (PSNP-DS) in Ethiopia and the Hunger Safety Net Programme (HSNP) in Nigeria were designed to address food insecurity. Leveraging on the success of these programmes, African governments, and external donors, mainly the World Bank, expanded CT programmes to address other challenges, including access to healthcare. Still, many CT programmes are geared towards relief or development goals [8]. Examples include the Zambia’s Child Grant Program (CGP) [14], the Subsidiary Reinvestment and Empowerment Program (SURE-P) in Nigeria [15], the Manicaland programme in Zimbabwe [16], the ‘Santé Nutritionnelle à Assise Communautaire dans la région de Kayes’ (SNACK – CAN) in Mali [17], among others [18].

CT programmes are on the rise after a wide proliferation during the COVID-19 pandemic [19]. CT programmes were a key strategy for recovery during the pandemic; 41% of overall social assistance programmes from 2020 to 2022 were CT programmes [20]. Within the pandemic period, an estimated 17% of the global population received support from CT programmes [21].

Findings about the effectiveness of these programmes in improving MCH outcomes, beyond nutritional outcomes and service utilisation remain inconclusive [22–24]. Most evaluations of these programmes tend to focus on the utilisation of healthcare services by the beneficiaries and other social determinants of health, thereby overlooking specific health outcomes related to mortality, individuals’ capacity to function, or the subjective sense of well-being [25]. The only synthesis we found examined mental health outcomes among young people under 25 years of age [26]. As suggested by R. G. Parrish [25], ‘positive health outcomes for people include being alive; functioning well mentally, physically and socially, and having a sense of well-being’. On this account, it could be argued that service utilisation is one underlying pathway through which CT programmes affect health outcomes. However, very few research syntheses have been done on these outcomes as the endpoints among women of childbearing age and children under 18 years of age. Several factors may be contributing to this gap. Many of the aforementioned studies lack a coherent operationalisation of the concepts of maternal and child health outcomes, beyond nutritional outcomes and service utilisation metrics [22,27,28], which creates larger heterogeneity across studies [23].

In this time of competing priorities across the world, the allocation of scarce resources needs to rely on evidence-based science. Both governments and external funders navigate resource constraints as they allocate funding. Lack of evidence on a given programmatic effort could undermine not only funding allocation but also any progress achieved. Hence, it is crucial to fill any potential gap in the existing literature regarding the effectiveness of CT programmes and their impact on MCH outcomes, to keep these programmes running, and to advocate in favour of scaling them to other geographies and demographics. Major barriers to the successful implementation and scale-up of these programmes include the lack of transparency, endemic corruption, and lack of valid data [29]. Yet, these limitations and issues represent opportunities for new areas of investigation.

Thus, this research synthesis focuses on the relationship between CT programmes and MCH outcomes, other than service utilisation, because these outcomes have been widely covered and synthesised about cash transfer programmes [27,30–32]. It covers studies that evaluated the effect/impact of CT programmes on MCH outcomes in sub-Saharan African countries for the period between 2000 and 2021. The focus on these countries stems from the fact that they are facing similar socioeconomic and political situations, and often share the same social norms, which are associated with populations’ health behaviours [33–36]. The nature and scope of CT programmes in sub-Saharan African countries differ from that of other LMICs. For instance, by contrast to Latin American countries whose CT programmes are solidarity-based nationwide social policies enacted by their respective governments, CT programmes in sub-Saharan African countries are often not an expression of deliberate policy programmes, with clear commitment by the national or local governments, except in some few countries (i.e. Ghana, Tanzania, Malawi, Kenya, and Zambia). Instead, CT programmes in those countries are driven by international agencies and donors who support small-scale pilot projects [37]. The feasibility of conducting a full systematic review assessing the impact of CT programmes on MCH outcomes other than service utilisation and nutritional outcomes would benefit from this scoping review that focuses on refining the conceptual breadth and years of coverage for such an effort in this region. Specifically, we will point to potential impact of CT programmes on MCH outcomes with limited or no evidence. The chosen period reflects the time when these programmes became available within the region [23].

This synthesis addressed the lack of evidence on the effectiveness of CT programmes to positively impact MCH outcomes, beyond service utilisation-based metrics and nutritional outcomes. Outcome indicators defined in this review were based on mortality, subjective health state, experiential and psychological state, and functioning. Protective behaviours that promote health were also considered. Hence, the following questions:

1. What is the impact of CT programmes to improve MCH outcomes (functional, subjective sense of well-being, experiential state, and death) in sub-Saharan African countries?
2. What are the potential pathways through which CT programmes influence MCH outcomes?

## Methodology

**Figure 1.**
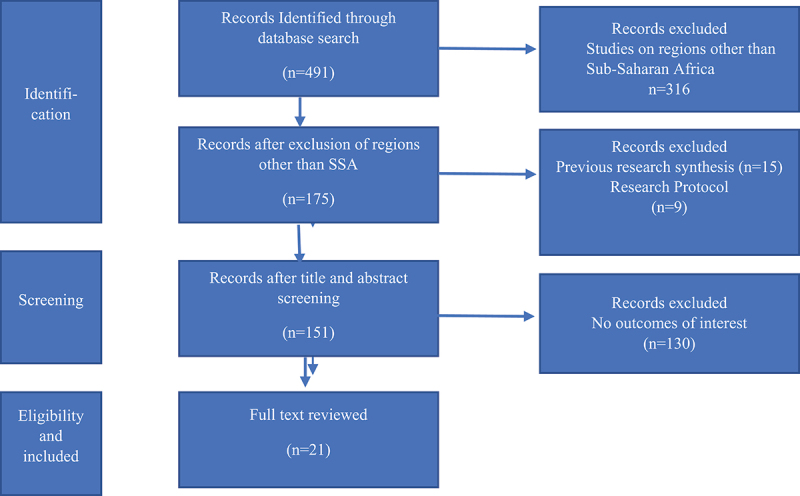
PRISMA flow chart for identification of studies.

We searched PubMed Central and Google Scholar for published peer-reviewed papers from 2000 to 2021. This research synthesis followed the Preferred Reporting Items for Systematic Reviews and Meta-Analysis (PRISMA) checklist (Figure 1).

Based on the above research questions, we used a combination of Medical Subject Headings (MeSH) and keywords, incorporating Boolean operators, truncation, and field tags. Our search string was defined as follows: (‘cash transfer*’ OR ‘cash incentive*’) AND (‘maternal and child health’ OR ‘maternal and child health outcome*’) AND ‘Africa’.

We also conducted a backward citation search, in which we looked at the reference list on the most recent and relevant systematic review, manually searching Google Scholar for relevant studies.

## Inclusion and exclusion criteria

Studies were screened for eligibility (i.e. experimental or quasi-experimental) and irrelevant articles were excluded based on title and abstract. Selected studies were fully reviewed for data extraction. The review included only peer-reviewed studies, whose CT programmes targeted women of reproductive age (15–49 years) and children (<18 years old) and reported health outcomes other than service utilisation. Studies of interest were implemented in the sub-Saharan African countries between 2000 and 2021.

Health outcomes included a measure of functional status (e.g. according to the International Classification of Functioning, Disability, and Health); a subjective sense of well-being, or mortality (survival). In addition, we included studies that reported some protective health behaviours (e.g. administration of nevirapine to prevent mother-to-child transmission of HIV).

## Data extraction and coding

Results from the search string in PubMed Central were exported in a text format file (i.e..txt) and saved in Excel. We first screened all the titles of articles to remove duplicates and studies that were conducted outside of the sub-Saharan African region, protocol, and systematic reviews. We then screen the abstracts of the remaining articles to further exclude irrelevant studies (e.g. studies not using the specified study population or did not report the defined health outcomes). Included articles were fully reviewed, and the following data were extracted: the name of the author, the year of publication, the title of the study, the study design, the name of the intervention if provided, the country of the intervention, the outcome assessed, the method used to evaluate impact, the reported measure of effect and the 95% confidence intervals or the standard errors, and the covariates. Since all studies were RCTs, they were evaluated for their quality, using the Consolidated Standard of Reporting Trials (CONSORT) 2010 checklist, and a risk of bias assessment was done using the revised Cochrane risk-of-bias tool for RCTs (RoB 2) (see table in appendix).

## Results

Final studies included five papers by the same group of authors evaluating a programme implemented in Malawi, and three papers from the same authors for a programme implemented in Kenya. Table 1 presents results from the synthesis by type of health outcome because many studies relied on the same dataset while investigating a diverse set of outcomes or endpoints (Table 1).

**Table 1.** Description of reported MCH outcomes by categories of health outcomes.

Outcome	Number of articles reporting
**Mortality**	**1 (4.8%)**
Child survival	1
Fetal loss/fetal death	1
Stillborn	1
Infant death	1
**Functional status**	**13 (61.9%)**
Illness in the past 30 days	3
Illness that stopped normal activities in the past 30 days	1
Cognitive, language, and motor growth	1
Depression	3
Children anthropometric measures	1
Incidence of HIV, HSV-2	2
**Well-being**	**3 (14.3)**
Subjective well-being	2
Mother’s general health	1
Happiness	1
Quality of life	1
**Health-promotion behaviours**	**4 (19.0)**
Prevention of mother-to-child HIV transmission	3
Tetanus toxoid vaccination	1
Protective sexual behaviors (e.g. condom use, etc.)	1

Almost all included studies were RCTs implemented in sub-Saharan African countries (i.e. Malawi, Kenya, South Africa, Nigeria, Tanzania, and Zambia). Assessed outcomes included: illness in the past 30 days or probability thereof, illness that stopped normal activities in the past 30 days, sexual debut, risky sexual behaviours (e.g. unprotected sex, multiple partners, transactional sex), depression, incidence of HIV and HSV-2, subjective sense of well-being, post-traumatic symptoms, sexual violence, child survival, fetal loss, fetal death, stillborn, infant death, child anthropometric measures, prevention of mother to child HIV transmission by the administration of nevirapine, and Early Infant Diagnosis of HIV (EID), happiness, well-being, child cognitive, language and motor development, child anthropometry.

Outcomes were grouped into three main groups based on the classification suggested by [25]: mortality, functional status, and subjective sense of well-being. In addition, an additional category was added to account for the relevance of some protective health behaviours reported.

Of the 21 articles included in the final review, health outcomes related to individual capacities to function were reported in 13 articles, representing 61.9% of the articles included in the review. Protective behaviours such as the administration of nevirapine to prevent mother-to-child transmission of HIV, etc. were reported in 19.0% (*n* = 4) of the articles. Three articles out of the 21 (14.3%) reported well-being outcomes, including subjective well-being, a mother’s general health, happiness, and self-reported quality of life. Only one article reported mortality or child survival outcomes.

### Mortality

**Figure 2.**
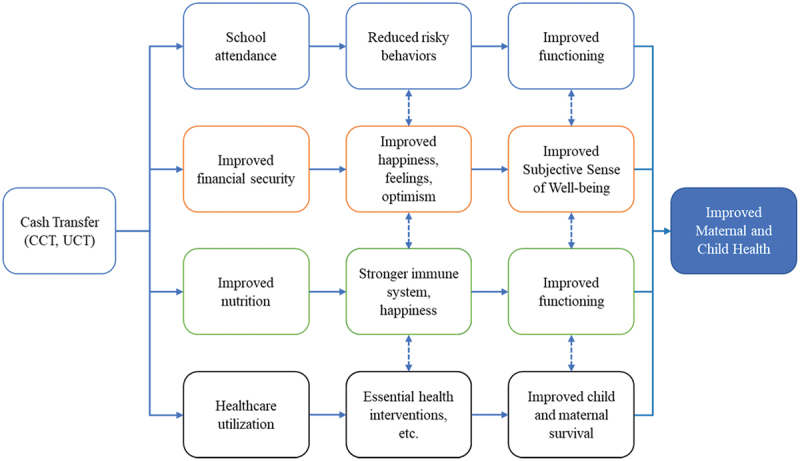
Pathways through which CT programs impact MCH outcomes.

The only article reporting mortality included outcomes on child survival, fetal death, stillborn, and infant death. The article reported a positive association between CCT programmes and child survival or reduced mortality (i.e. a child who was in utero at enrolment was alive at follow-up). In Nigeria, a CCT programme was associated with substantial increases in child survival (ITT: 0.0606; std. error: 0.0098, *p*-value < 0.01). The increase in overall child survival was driven by a large decrease in fetal losses (29%-point decrease in the treatment group relative to the control group mean). Potential causal mechanisms included conditionalities attached to a cash payment of $14 (i.e. if eligible pregnant women used a package of health services consisting of at least three antenatal care visits, a health facility delivery, and one postnatal visit). Provision of other essential health interventions (e.g. antenatal care, immunisation, screening for childhood pneumonia, etc.) at the beginning of life, are also key mechanisms that could improve child survival (Figure 2).

### Functioning outcomes

Specific outcomes included episodes of illness (reported in four articles), assessment of depressive symptoms (reported in five articles), and incidence of HIV and HSV-2 (reported in two articles). CT programmes impacted these outcomes, mainly through school attendance [38,39], and also through financial and food security [40,41].

Evidence emerged through these studies that CCT was associated with improved health outcomes. For instance, participants who received treatment (cash payment) reported reduced likelihood of illness episodes in the past 30 days (e.g. OR: 0.67; 95% C.I.: 0.44–0.90), reduced likelihood of reporting difficulties in performing normal activities due to illness (e.g. OR: 0.58; 95% C.I.: 0.40–0.82). Lack of CCT was associated with higher school dropouts, which in turn were associated with a higher risk of HIV (HR: 2.97; 95% C.I.: 1.62–5.45) and HSV-2 (HR: 2.47; 95% C.I.: 1.46–4.14). UCT was associated with a lower likelihood of reporting the presence of depressive symptoms (coef.: −2.05; std. error: 0.475; *p*-value < 0.001).

### Well-being

Three studies reported outcome measures related to participants’ well-being. Those included subjective sense of well-being, general health, happiness, and self-reported quality of life. For instance, K. Kilburn, S. Handa, G. Angeles, M. Tsoka and P. Mvula [42] found that UCT programmes were associated with improved quality of life (e.g.: coef.: 3.18, *p*-value < 0.05). In addition, other studies reported positive effects of UCT on a few subjective outcomes such as self-reported overall satisfaction with life (e.g. Intention to Treat (ITT): 0.673, *p*-value < 0.001), my child enjoys life (ITT.: 0.169 *p*-value < 0.001), I feel positive about my child’s future (ITT.: 0.202, *p*-value < 0.001).

### Behavioral and protective interventions

Although the main focus of the study was to synthesise the existing literature concerning the three outcome categories suggested by R. G. Parrish [25], we expanded the review to accommodate protective behaviour promoting health. These outcomes are important given their protective nature and the risk associated with the lack thereof. The present review captured protected sex, tetanus toxoid vaccination, and health visits for nevirapine intake to prevent mother-to-child transmission of HIV infection. Studies reported a positive effect of CCT programmes on the incidence of HIV and other sexually transmitted infections (STIs) through behavioural mechanisms such as condom use, reduced number of sexual partners, reduced incidence of transactional sex, and administration of nevirapine to prevent mother-to-child transmission of HIV infection. Another study looked at the reception of tetanus toxoid vaccination during the perinatal period. The study showed that CCT programmes were associated with an increased likelihood of receiving the vaccine and therefore of being protected against maternal and neonatal tetanus (OR for those who received C300: 3.362; 95% C.C.: 2.595–4.354; and for those who received C800: 7.575; 95% C.I.: 5.648–10.160).

## Discussion

This review synthesises evidence on the impact of CT programmes on MCH outcomes other than service utilisation, in sub-Saharan African countries. Although it was initially designed to address nutritional needs in Latin American countries [43], CT programmes have gained momentum around the world, including among African countries.

A lack of conclusive evidence on the effectiveness of CT programmes in addressing MCH outcomes other than service utilisation persists. This synthesis provides evidence that CT programmes effectively improve MCH outcomes beyond nutritional outcomes and service utilisation. For instance, we found that CCT programmes are effective in averting fetal and infant deaths and improving child survival. Our synthesis also found that CCT programmes are not only effective but they are also cost-effective in preventing mother-to-child transmission of HIV infection in limited resource settings [44]. This synthesis suggested that the conditionalities attached to most of the CCT programmes also represent pathways through which CT programmes influence health outcomes [8,9]. The review showed that CCT programmes are effective in improving child survival, functional health statuses, and promoting healthy behaviours, whereas UCT are mainly effective for impacting measures of well-being. This finding carries significant policy implications for future interventions. The conditionalities attached to some CT programmes are important reinforcing mechanisms that could promote the performance of healthy behaviours, whereas unconditional cash transfer could target the most deprived segment of the population to improve their well-being, through the provision of social support (e.g. financial security, food, etc.).

For instance, the provision of essential healthcare interventions (e.g. screening for anemia, prophylactic antibiotics for cesarean section, exclusive breastfeeding, case management of diarrhea, or childhood pneumonia, etc.) to deprived populations early on in life is one mechanism through which CCT programmes impact children’s survival. Consistent with the literature on life course perspective [45,46] every year, an estimated 6.54 million children die under the age of 5 and the child’s risk of death is the highest during the first 28 days of life [47,48]. However, financial constraints (e.g. fees for healthcare services, transportation, payment of medicines, etc.) and cultural beliefs remain significant barriers to healthcare access in most of these countries [49,50]. Hence, providing financial incentives to encourage women and their children to benefit from those interventions during critical moments (e.g. gestational period, post-delivery, and early childhood) has a significant impact on their survival and functional experiences. However, the performance of this behaviour could be modified by a lack of motivation and other cognitive factors. Therefore, attaching conditionality to such programmes can contribute to improving adherence to promoted behaviours.

The synthesis also found evidence that CCT programmes were associated with improved functioning capacities (e.g. lack of illness in the past 30 days; no report of being unable to perform usual activities because of illness, reduced depressive symptoms, and incidence of HIV and HSV-2). Across studies, there seems to be a consensus that CCT programmes impact these outcomes through two underlying mechanisms, namely education [38,39] and financial and food security [17,23]. CCT programmes are used to keep young children, especially young girls, in school, which reduces their likelihood of engaging in risky behaviours (e.g. transactional sex, unprotected sex, etc.). In addition, CCT programmes are also used to provide financial resources to deprived populations. Thus, recipients of these grants can use the money to buy food, thereby preventing food insecurity which might compromise their functional capacities [51]

UCT programmes were associated with improved emotional health (e.g. subjective sense of well-being, self-reported quality of life). UCT programmes impact people’s sense of well-being, by providing financial support to people whose lives are subjected to daily concerns over the basic needs of food, shelter, clothing, etc. Given that emotions influence health directly (e.g. through the activation of the hypothalamic-pituitary-adrenal axis) and indirectly (e.g. through health behaviours), fewer episodes of stress imply improved health among participants [52]. For instance, a study reported that the UCT programme was associated with positive feelings about children’s future, and generally happiness among children in recipient households [53].

Although we found supporting evidence of the positive impact of these CT interventions on MCH outcomes, it should be underscored that one of the key issues plaguing the implementation of these programmes is the lack of transparency and auditability at all levels [29,54]. For instance, the decision on where to implement these programmes (e.g. selecting a given state or province rather than others), and who should benefit from such programmes cast questions on its transparency, and accountability. Quality data and information are fundamental for the effective implementation and evaluation of programmes [55]. The lack thereof exposes the programme to shortcomings (e.g. corruption, lack of transparency and accountability), and to missed opportunities for improving its robustness. In the context of CT programmes, data collection remains a very expensive and often complex operation, plumbing their effective implementation.

To optimise data collection processes, countries may need a tamper-proof system that provides security, privacy, confidentiality, and most importantly, decentralisation of data collection activities [56]. The relevance of this approach in the context of sub-Saharan African countries stems from the fact that most of these countries rely on international aid, often tied to stringent conditionalities, to implement, and sustain such programmes [57]. When the lack of transparency and accountability becomes an issue, most of the external donors become hesitant to fund the programme. Hence, given the abovementioned evidence on the effectiveness of CT programmes in improving MCH outcomes, it is crucial to improve the implementation of these programmes to ensure current and potential stakeholders regarding the actual use of the funds and the impact thereof.

## Limitations

The scope of this scoping review was to synthesise the existing literature in a rather nascent field. To avoid duplication of existing literature, we limited our analyses to exclude service utilisation and nutritional outcomes. As a result, outcomes commonly considered to be associated with MCH outcomes (e.g. prenatal care visits) were excluded and across several domains of outcomes defined in this synthesis, there were only one or two existing studies in which to analyse the findings. Further research synthesis should expand this research to draw more statistical conclusions, not only in sub-Saharan African countries but across LMICs. Studies should consider using a standardised framework for assessing health outcomes, such as the one proposed by R. G. Parrish [25]. Also, the International Classification of Functioning, Disability, and Health could be used to inform the selection and reporting of measures of functional status. We were unable to analyse the rise of CT programmes post-pandemic as our review ended in 2021; with the changes because of the pandemic itself and a growing acceptance of CT programmes, future studies should evaluate the results of CT programmes after 2021.

## Conclusions

We found compelling evidence that in sub-Saharan African countries, CT programmes are effective interventions with a positive impact on MCH outcomes, including survival experience, functional status, and emotional health. The quality of evidence is higher because most studies were randomised controlled trials. The underlying mechanisms through which CT programmes operate have been discussed. Policymakers and funders should tailor such interventions based on the mechanisms discussed. However, more needs to be done to improve transparency in the implementation of these programmes.

## Paper context

Cash transfer programmes have been expanded in many African countries and have shown promising results. However, their impacts on maternal and child health remain inconclusive, beyond service utilisation and nutritional outcomes. This paper proposes a framework for reporting such health outcomes and discusses potential mechanisms through which these programmes can be used to improve maternal and child health outcomes. The paper also suggests instances when conditionalities are needed to improve adherence.

## Appendix List of studies included for full review

**Table:**

Author(s)	Year	N (Sample)	Target Population	Exposure, Intervention	Outcome(s) assessed	Methods	Confounders
Akresh, R.; De Walque, D.; Kazianga, H.	2016	–	Young Children (under 5)	Cash transfers	C-reactive protein (CRP) Probability of being ill Anthropometric indicators	Multilevel linear regression models	Village characteristics (e.g. distance to province capital, source of water, rainfall, presence of a village market) Health facility characteristics (facility offers nutritional counseling/supplement/vaccinations, clinic funding sources, and recent village history of health epidemics)
Berk Özler, Kelly Hallman, Marie-France Guimond, Elizabeth A. Kelvin, Marian Rogers, Esther Karnley	2020	Liberia (*N* = 999 men aged 18–35)	Young girls 13–14	Cash transfer	Sexual violence, sexual and reproductive health, psychosocial wellbeing	ITT, using a linear regression model	age
Sylvia Shangani, Don Operario, Becky Genberg, Kipruto Kirwa, Miriam Midoun, Lukoye Atwoli, David Ayuku, Omar Galárraga, Paula Braitstein	2017	*N* = 655	10–18 years orphans and vulnerable adolescents	Unconditional cash transfer	Depression Anxiety Post-traumatic stress symptoms	Logistic regression models	Age, sex, orphan status, enrolled in school, number of siblings, deceased siblings, religion important, pair of shoes Medical (hospitalised in the past year) sexually abused, transactional sex. Caregiver characteristics
Yesim Tozan, Sicong Sun, Ariadna Capasso, Julia Shu-Huah Wang, Torsten B. Neilands, Ozge Sensoy Bahar, Christopher Damulira, Fred M. Ssewamala	2021	*N* = 1,383	Young people (10–16) in Uganda	Cash transfer through saving incentives	Self-rated health Sexual health (sexual risk-taking intention, HIV prevention attitudes, HIV knowledge) Mental health functioning (depression, hopelessness, self-concept, self-efficacy)	ITT, using multilevel models	Age, sex, wave of data collection
Winnie K Luseno, Kavita Singh, Sudhanshu Handa, Chirayath Suchindran	2014	1197	Children 6–17 years	Cash transfer program to address poor school-age children’s health outcomes (4 village groups were randomly selected to receive cash transfers and 4 others did not). The amount of cash depended on the household size. **CCT**	Illness in the past month Illness that stopped normal activities in the past month Missing school due to illness or injury in the past month Health care use for worst illness in past year	Multilevel logistic regression model	Household-level variables (e.g. number of working-age adults, number of sick adults in past 30 days, age head of household, sex head of household (HH), education head of household, number of children) Child-level variables (maternal orphan, paternal orphan, double orphan, child sex) control variables (age, relationship to HH, control for outcome at baseline)
Sudhanshu Handa, Carolyn Tucker Halpern, Audrey Pettifor, Harsha Thirumurthy	2014	–	Orphans Vulnerable Children (OVC), aged 0–17 years	Unconditional cash transfer of $20 per month to caregivers of young people. **UCT**	Sexual debut Sexual behaviours (e.g. condom use, number of partners, transactional sex)	Logistic regression model	Age of head, sex of head, education of head, relationship of child to respondent, residence, sex of child, age of child
Gustavo Angeles, Jacobus de Hoop, Sudhanshu Handa, Kelly Kilburn, Annamaria Milazzo, Amber Peterman	2019	3531 SCTP-eligible households 2782 youth	13–19	Unconditional cash transfer **UCT**	10-item short form of CES-D scale Depressive symptoms	Intention-to-treat using ANCOVA & Difference in difference for sensitivity analysis	Baseline youth characteristics (age, single or double orphan), baseline household characteristics (sex of caregivers, literacy status, household size) traditional authority ID
Carolyn Huang, Kavita Singh, Sudhanshu Handa, Carolyn Halpern, Audrey Pettifor, Harsha Thirumurthy	2017	0–7 years and under 5 years of age		Unconditional cash transfer of $20 per month to caregivers of young people	Incidence of illness (malaria/pneumonia) Health seeking behaviors	Generalized Linear Latent and mixed method estimation	Individual level (age, sex, orphan, relationship to HH, low Haz, low BMIZ) Characteristics of the head of household (education, age, sex). Environmental factors (household size, living environment index, crowding index, cook stove, cook fuel, toilet, diet diversity score, food groups as a proportion of diet)Wealth: monthly per capita income, livestock indexCommunity characteristics (distance malaria treatment, distance doctor, residence: rural/urban)
Kelly Kilburn, Harsha Thirumurthy, Carolyn Tucker Halpern, Audrey Pettifor, Sudhanshu Handa	2016	2,210	15–24 young persons	**UCT** (CT-OVC)	CES-D (Depression Index)	Logistic regression model (estimating Presence of depressive symptoms)	Age, sex, participant’s relationship to head of HH, head of household characteristics (sex, age, schooling attainment), Nairobi residence status, indicator of morbidity status (except for physical outcomes)
Marie CD STONER, Audrey PETTIFOR, Jessie K. EDWARDS, Allison E. AIELLO, Carolyn T HALPERN, Aimée JULIEN, Amanda SELIN, Rhian TWINE, James P. HUGHES, Jing WANG, Yaw AGYEI, F. Xavier GOMEZ-OLIVE, Ryan G. WAGNER, Catherine MACPHAIL, Kathleen KAHN	2018	2,328	13–20 years	CCT conditional on school attendance	Incidence of HIV and HSV-2	Inverse probability-of-exposure weighted survival curves and a marginal structural Cox model.	Age, household wealth, ever repeated a grade, partner in 5 years or older, double, or single orphan, maximum age difference with partners, ever had sex, unprotected sex in last 3 months, given money or gift in exchange for sex, alcohol use, children depression index, children manifest depression index
Kelly Kilburn, Sudhanshu Handa, Gustavo Angeles, Maxton Tsoka, Peter Mvula	2018		Ultra-poor population (identified by appointed community leaders)	**UCT**	Subjective Well-being (SWB): QoL, future expectations, and relative well-being	Difference in Difference regression model	Control variables included individual and household characteristics plus a full set of treatment-covariate interactions
Edward N. Okeke, Isa S. Abubakar	2020	10582 women	Pregnant women	CCT	Child survival, fetal loss, fetal death, stillborn, infant death (neonatal vs post-natal death)	ITT	dummies for mother’s age (<18, 18–24, 25–29,30–34, and>35 years), dummies for mother’s educational attainment (no schooling, Islamic schooling, some primary school, some secondary school, and some tertiary schooling), a dummy denoting Hausa or Fulani extraction, dummies for mother’s number of prior births, dummies indicating a prior fetal loss or a stillbirth, and household wealth quintile dummies
Jenny X. Liu, Jennifer Shen, Nicholas Wilson, Svetha Janumpalli, Patrick Stadler, Nancy Padian	2019	554	Pregnant women tested HIV positive	PMTCT	Administration of nevirapine, administration of EID	ITT	
Nadia Nguyen, Kimberly A. Powers, William C. Miller, Annie Green Howard, Carolyn T. Halpern, James P. Hughes, Jing Wang, Rhian Twine, Xavier Gomez-Olive, Catherine MacPhail, Kathleen Kahn, Audrey E. Pettifor	2019	1,034	Young girls 13–23	Sexual Partners Anonymous out-of-school partner	Incidence of HIV	Latent Class Analysis	
Luisa Natali, Sudhanshu Handa, Amber Peterman, David Seidenfeld, Gelson Tembo, On behalf of the Zambia Cash Transfer Evaluation Team	2018		Women who are primary caregiver of eligible child	UCT	Happiness Child’s wellbeing	Linear probability	woman’s age, education and marital status, household size and household demographic composition, and districts. Each outcome in column 3–12 reflects the woman’s level of agreement with each of these statements measured using a five-point Likert Scale ranging from one to five (where 1 capture strong disagreement and 5 strong agreement)
Edward N. Okeke, Zachary Wagner, Isa S. Abubakar	2020	180 Primary Health Facility/1.2M people	Pregnant women	CCT to deliver in hospitals	Mother’s General Health Delivery complications	ITT	
Christopher R Sudfeld, Lilia Bliznashka, Geofrey Ashery, Aisha K Yousafzai, Honorati Masanja	2021	12 Villages	Pregnant women with child < 1 year	Community Health Worker (CHW) alone or CHW with CCT	Child Cognitive, language and motor development (Bayley scale) Child length/height-for-age-z-score (HAZ)	ITT	
Jessica Londeree Saleska, Abigail Norris Turner, Maria F. Gallo, Abigail Shoben, Bienvenu Kawende, Noro Lantoniaina Rosa Ravelomanana, Harsha Thirumurthy, Marcel Yotebieng	2021	434 HIV pregnant women	HIV positive women	CCT	Retention in HIV care Uptake of prevention of mother-to-child transmission services	Binomial models	Wealth index, depression symptoms, alcohol use in pregnancy
Yousafzai, Aisha K1 (AUTHOR), Asheri, Geofrey2 (AUTHOR), Masanja, Honorati2 (AUTHOR), Sudfeld, Christopher R1,3 (AUTHOR)	2020	12 villages	Pregnant women and children	CHW, CHW + CCT	Maternal depressive symptoms	Hopkins Symptoms Check List 25 (HSCL-25)	age, pregnancy status at enrollment, education, parity, marital status, perceived social support, household wealth, child age
Angeles, Gustavo, de Hoop, Jacobus, Handa, Sudhanshu, Kilburn, Kelly, Milazzo, Annamaria, Peterman, Amber	2019	330,000 households		CCT	Child anthropometry	10-items short form of CES-D scale	
Ryoko Sato and Benjamin Fintan	2019	2,530 Women 15–35	Women 15–35 years	CCT	Tetanus toxoid vaccination	Logistic regression model	Sociodemographic, distance to clinic, transport to clinic

Risk of bias assessment (Intention-to-treat).
